# Correction: Perylene-derivative singlet exciton fission in water solution

**DOI:** 10.1039/d4sc90232g

**Published:** 2024-12-04

**Authors:** Chloe Magne, Simona Streckaite, Roberto A. Boto, Eduardo Domínguez-Ojeda, Marina Gromova, Andrea Echeverri, Flavio Siro Brigiano, Minh-Huong Ha-Thi, Marius Franckevičius, Vidmantas Jašinskas, Annamaria Quaranta, Andrew A. Pascal, Matthieu Koepf, David Casanova, Thomas Pino, Bruno Robert, Julia Contreras-García, Daniel Finkelstein-Shapiro, Vidmantas Gulbinas, Manuel J. Llansola-Portoles

**Affiliations:** a Université Paris-Saclay, CEA, CNRS, Institute for Integrative Biology of the Cell (I2BC) Gif-sur-Yvette 91190 France manuel.llansola@i2bc.paris-saclay.fr; b Department of Molecular Compound Physics, Center for Physical Sciences and Technology Saulėtekio Avenue 3 Vilnius LT-10257 Lithuania; c Donostia International Physics Center (DIPC) Donostia 20018 Basque Spain; d Insituto de Química, Universidad Nacional Autónoma de México Mexico City 04510 Mexico; e Université Grenoble Alpes, CNRS, CEA, IRIG, MEM Grenoble F-38054 France; f Sorbonne Université, CNRS, Laboratoire de Chimie Théorique, LCT Paris F-75005 France; g Université Paris-Saclay, CNRS, Institut des Sciences Moléculaires d'Orsay Orsay 91405 France; h Université Grenoble Alpes, CNRS, CEA, IRIG, Laboratoire de Chimie et Biologie des Métaux Grenoble F-38054 France; i IKERBASQUE, Basque Foundation for Science Bilbao 48009 Basque Spain

## Abstract

Correction for ‘Perylene-derivative singlet exciton fission in water solution’ by Chloe Magne *et al.*, *Chem. Sci.*, 2024, **15**, 17831–17842, https://doi.org/10.1039/D4SC04732J.

The authors regret that the name of one of the authors of this article (Marius Franckevičius) was misspelled in the originally published version. The corrected author list can be found above.

The Royal Society of Chemistry apologises for these errors and any consequent inconvenience to authors and readers.

